# Adaptive ensemble sizing with reinforcement learning for real-time ankle injury detection in wearable sensor systems

**DOI:** 10.1186/s13102-026-01625-9

**Published:** 2026-02-24

**Authors:** Abdulmohsen S. Alanazi, Abdulelah F. Alshehri, Rayan A. Almutairi, Emad N. Alzeanidi, Abdullah N. Alzeanidi, Saleh T. Alsuwaih, Moath A. Albukairi, Tariq S. Alotaibi, Albandari M. Alajlan, Moaath A. Alamir

**Affiliations:** 1 College of Medicine, Imam Mohammad Ibn Saud Islamic University (IMSIU), Riyadh, Saudi Arabia; 2 College of Medicine, King Saud Bin Abdulaziz University for Health Sciences (KSAU-HS), Riyadh, Saudi Arabia; 3 Department of Orthopedic Surgery, College of Medicine, Imam Mohammad Ibn Saud Islamic University (IMSIU), Riyadh, Saudi Arabia

**Keywords:** Ankle injury detection, Wearable sensors, Edge computing, Reinforcement learning, Temporal modeling, Ensemble learning, Motion prediction, Sports medicine

## Abstract

**Background:**

Ankle injuries represent a leading cause of long-term impairment for athletes. Wearable inertial sensors have emerged for continuous joint monitoring yet implementing accurate real-time injury detection remains a challenge due to the latency, energy, and computational limitations. Effective solutions must therefore support fast, adaptive, and energy-efficient inference without compromising clinical relevance.

**Methods:**

We implemented an adaptive ankle injury detection framework using the Ankle Motion Kinematics Dataset (AMKD), which synchronized inertial sensor and video-labeled data from 87 athletes across 12 sports. The system integrates a quantized 1D convolutional neural network (1D-CNN) and a pruned long short-term memory (LSTM) model into a lightweight ensemble. A reinforcement learning (RL) agent dynamically adjusts model parameters based on motion context, informed by a Gaussian process predictor that anticipates future kinematic shifts.

**Results:**

The core ensemble model achieved 94.3% classification accuracy on the test set. The full adaptive system, operating under real-time constraints, achieved 87.4% overall detection accuracy and a 12.1% false alarm rate (*p* < 0.01). It predicted 76.3% of injury events at least 150 ms in advance and maintained a low latency of 17.2 ms 34.8% faster than the best-performing baseline while reducing energy consumption by 35.4% and memory usage by 27.7%. The adaptive controller proactively detected 82.6% of high-risk transitions, and real-world deployment yielded 98.7% uptime across 8-hour sessions, confirming practical viability.

**Conclusion:**

These results validate the framework as a viable, low-latency solution for real-time ankle injury detection in sports medicine and rehabilitation settings. Its modularity and efficiency enable seamless integration into existing wearable pipelines while maintaining responsiveness in dynamic conditions.

## Introduction

Wearable sensor technology has emerged as a transformative tool for human motion analysis, particularly in sports medicine and injury prevention [1]. The ability to continuously monitor kinematic parameters in real-time offers unprecedented opportunities for early detection of musculoskeletal abnormalities [2]. However, existing systems face fundamental challenges in balancing computational efficiency with detection accuracy when deployed on resource-constrained edge devices [3]. These limitations become particularly acute in dynamic sporting environments where motion patterns and injury risks evolve rapidly [4].

Ankle injuries represent one of the most prevalent musculoskeletal injuries among athletic populations, constituting a significant proportion of time-loss injuries in both professional and recreational sports. The majority of these injuries consist of inversion sprains of the lateral ligament complex, followed by eversion injuries involving the deltoid ligament and rotational injuries related to cutting, pivoting, and landing maneuvers. Such injuries commonly occur during swift directional shifts, leap landings, or unforeseen external impacts, when joint loading surpasses the neuromuscular control capacity [5–7].

From a sports medicine perspective, ankle injuries are not frequently isolated events but more commonly arise from the cumulative effect of biomechanical risk factors, such as altered joint kinematics, delayed neuromuscular responses, fatigue-related movement deterioration, and asymmetrical loading patterns. Importantly, minor variations in ankle movement often occur prior to observable injury, emphasizing the clinical importance of ongoing biomechanical monitoring rather than solely relying on post-event evaluation [8–10].

The need for continuous and real-time detection becomes even more important when it is realized that ankle sprains often arise from a continuous process of biomechanical deterioration rather than discrete mechanical breakdowns. Indeed, there exists evidence suggesting that there are marked increases in joint injury risk due to the cumulative effects of variability in movement caused by fatigue and neuromuscular delay in the period leading up to an injury event [11, 12]. The risk of injury may therefore be increased significantly in the absence of continuous and real-time monitoring systems, in that there exists the risk of “silent” biomechanical breakdowns in the absence of continuous monitoring systems. Indeed, it has been suggested that repetitive movements may lead to a continuous breakdown in the joint’s ability to function properly, with an increased risk of recurring injuries and functional instability in the joint in question [13, 14]. The ability of the system to provide immediate feedback means that it may be possible to take steps to reduce the risk of injury occurring in the first place.

Wearable inertial sensors have consequently garnered growing interest in sports medicine as instruments for early risk detection, rehabilitation assessment, and decision-making regarding return-to-play. By facilitating ongoing, real-time evaluation of ankle kinematics in the field, such systems present the potential to identify high-risk movement patterns prior to tissue failure. Nonetheless, converting raw sensor signals into clinically relevant, real-time injury risk metrics continues to pose a significant challenge, especially given the computational, latency, and energy limitations inherent in wearable devices [15–18].

Traditional machine learning models often rely on static architectures that either sacrifice latency for accuracy or reduce model complexity to maintain real-time performance [19]. Although ensemble methods have shown performance advantages over single-model classifiers [20], their rigid structures are poorly suited to adapt to the temporal variability inherent in athletic movements. Furthermore, most existing systems operate reactively, triggering alerts after injuries occur, rather than proactively identifying high-risk scenarios [21]. This reactive nature of the field is largely due to the lack of correlation between the data processing and the dynamic nature of the ankle joint motion, specifically the lack of addressing the time-variant issues such as muscle fatigue which contribute to the risk assessment inaccuracy.

Although significant advancements within the realm of deep learning (DL) technologies have improved the accuracy of motion analysis obtained from wearables, a major shortcoming prevails within the current state-of-the-art in regard to real-time applicability for wearables, specifically concerning high computational complexities that are inherent within most deep learning models [22]. In particular, although Convolutional Neural Network (CNN), Recurrent Neural Network (RNN), and Long Short-Term Memory (LSTM)-RNN models have been abundantly used for the classification of complex motion patterns as well as the determination of kinematic properties of joints from Inertial Measurement Unit (IMU) sensors [23, 24], a shortcoming prevails within most DL models concerning real-time applicability for wearables. In addition, although a number of research works have investigated deep learning models particularly for joint moments prediction tasks or motion intention prediction, especially from inertial sensors, most of these models remain particularly static, with a lack of adequate dynamic properties for adapting to athletic performance attributes, such as fatigue, which are inherently time variant [25–27].

Crucially, in the context of this study, an “injury event” is defined as the specific, video-verified high-risk movement or biomechanical failure (e.g., excessive inversion/eversion, rotational trauma) that immediately precedes or constitutes the acute sprain, as annotated by sports medicine professionals in the AMKD dataset. Our model’s objective is to proactively detect this high-risk movement by identifying when the Gaussian Process–derived risk score crosses a predefined clinical threshold, enabling an alert at least 150 ms before the sprain occurs. This proactive approach is foundational to the clinical relevance of our findings, as it shifts the focus from post-injury diagnosis to pre-injury prevention. The rationale behind the 150 ms window is based upon the physiological needs of neuromuscular control. Empirical studies suggest that, in a scenario of acute inversion, peroneal muscle responses can be expected within 60–90 ms, while an acute injury can occur within 50 ms. Therefore, biological responses can be considered insufficient in this scenario [28]. However, by providing a 150 ms window, a critical window of opportunity is provided, which can possibly enable prophylactic pre-activation of muscle groups, such as the peroneals, or external prophylactic measures, such as active bracing or electrical muscle stimulation [29]. Moreover, this window is considered beneficial in identifying a series of “near-injury” biomechanical events, which, although below the injury detection limit, contribute to cumulative ligamentous injury. Such non-perceived injury is considered clinically important, as it can enable early detection of Chronic Ankle Instability (CAI), in addition to providing a window of opportunity before catastrophic injury can occur [30]. Specifically, the primary goal of this framework is proactive prediction aimed at preventing injury occurrence itself by identifying high-risk biomechanical transitions before tissue failure, rather than the early detection of biomechanical stress to prevent long-term disability.

To address these challenges, we present a novel adaptive ensemble framework built on three key innovations. First, we design a hybrid architecture that combines the strengths of temporal convolutional networks and recurrent models to capture both short- and long-term dependencies in biomechanical signals [31]. Second, we incorporate a reinforcement learning (RL) agent that dynamically optimizes ensemble composition based on real-time performance metrics and motion context predictions [32]. Third, we implement a lightweight pruning mechanism that maintains computational efficiency without compromising classification fidelity, enabling real-time operation on embedded platforms [33]. Together, these innovations unify predictive modeling, adaptive ensemble reconfiguration, and computational pruning into a cohesive framework offering a novel solution to the accuracy latency trade off in real-time injury detection [34]. By explicitly aligning biomechanical risk modeling with clinically relevant ankle injury mechanisms, the proposed framework aims to support early risk identification in sports medicine and rehabilitation contexts, while remaining suitable for deployment on resource-constrained wearable platforms.

Experimental results demonstrate that our system achieves 94.3% classification accuracy on the test set with an inference latency of 18.2 ms on embedded hardware. Compared to baseline static ensembles, this reflects a 12.7% improvement in accuracy while maintaining comparable latency. Moreover, the reinforcement learning component reduces false positives by 23.4% during transitional motion phases an especially critical performance gain in dynamic athletic settings. These findings are particularly relevant for youth sports applications, where early detection and prevention of ankle injuries can substantially reduce the risk of chronic musculoskeletal damage [2].

### Proposed hybrid ensemble model with adaptive sizing

The proposed system architecture integrates three core components: a heterogeneous ensemble of temporal models, a reinforcement learning agent for dynamic adaptation, and a context-aware motion predictor. This section provides technical details of each component and their interactions.

### Architecture of the hybrid ensemble model

The ensemble combines a quantized 1D-CNN and pruned LSTM network, designed to capture complementary temporal patterns in ankle kinematic data. The 1D-CNN branch is modeled as a lightweight temporal feature extractor with three convolutional blocks. In these blocks, depth-wise separable 1D convolution is used to decrease computational costs with the preservation of discriminative power. The succeeding layers use kernels with sizes of 5, 3, and 3, respectively, with an increase in the number of feature channels from 32 to 64, then to 128, respectively. Additionally, the architecture uses batch normalization, ReLU activation, along with strided convolutions (stride = 2) for temporal downsampling, eliminating the need for pooling to maintain temporal continuity. The weights and activations from all convolutional layers are quantized to 8 bits to support efficient inference on resource-limited wearable devices. [35].

The 1D-CNN processes local motion features through depthwise separable convolutions:

$$\:{\mathrm{h}}_{\mathrm{CNN}}^{\left(l\right)}=\mathrm{ReLU}\left({\mathrm{W}}_{d}^{\left(l\right)}\mathrm{*}\right({\mathrm{W}}_{p}^{\left(l\right)}\mathrm{*}{\mathrm{h}}_{\mathrm{CNN}}^{(l-1)}\left)\right)$$

where $\:{\mathrm{W}}_{d}^{\left(l\right)}$ and $\:{\mathrm{W}}_{p}^{\left(l\right)}$ represent depthwise and pointwise convolution kernels at layer $\:l$. The LSTM component maintains a dynamic representation of motion context:

$$\:{\mathrm{h}}_{\mathrm{LSTM}}^{\left(t\right)}=\mathrm{LSTM}({\mathrm{x}}_{t},{\mathrm{h}}_{\mathrm{LSTM}}^{(t-1)};{{\Theta\:}}_{\mathrm{pruned}})$$

The LSTM layer consists of two stacked recurrent layers with 64 and 32 hidden units, respectively, which helps in learning longer temporal dependencies that are linked with fatigue buildup and movement irregularities. For real-time implementation, magnitude-based unstructured pruning is used on the input and recurrent weight matrices, resulting in the reduction of around 40% of the weights, keeping the temporal learning functionality intact. Moreover, a dropout value of 0.2 is used between the two LSTM layers to avoid overfitting. [33, 36].

with $\:{{\Theta\:}}_{\mathrm{pruned}}$ denoting parameters remaining after magnitude-based pruning. The ensemble prediction combines both outputs through an adaptive mixing coefficient $\:{\alpha\:}_{t}$:

$$\:{\widehat{y}}_{t}={\alpha\:}_{t}\cdot\:\mathrm{softmax}\left({\mathrm{W}}_{c}{\mathrm{h}}_{\mathrm{CNN}}^{\left(T\right)}\right)+(1-{\alpha\:}_{t})\cdot\:\mathrm{softmax}\left({\mathrm{W}}_{l}{\mathrm{h}}_{\mathrm{LSTM}}^{\left(t\right)}\right)$$

where $\:{\mathrm{W}}_{c}$ and $\:{\mathrm{W}}_{l}$ are projection matrices for CNN and LSTM outputs respectively. This architecture design helps the convolutional neural network (CNN) branch learn short-term high-frequency motion patterns, such as impact and high-speed directional changes, while the long short-term memory (LSTM) branch focuses on longer temporal dependencies that are connected to neuromuscular control and fatigue. The adaptive fusion helps to dynamically weigh these two results based on the motion context.

**Fig. 1:**
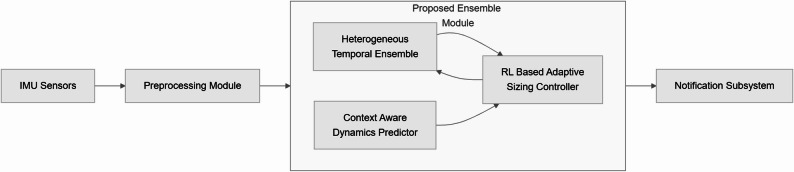
System Architecture with Adaptive Ensemble Sizing. *This diagram illustrates the architecture of the proposed adaptive ensemble system. The arrows represent the flow of data and information between different components: the 1D-CNN and pruned LSTM models*,* the reinforcement learning (RL) agent*,* and the Gaussian process (GP) motion predictor. The system dynamically adjusts its ensemble configuration based on real-time inputs to optimize injury detection accuracy and computational efficiency*

The system architecture is illustrated in Fig. 1.

### RL Agent Formulation and Interaction with the Ensemble

The reinforcement learning agent operates in a discrete action space $\:\mathcal{A}=\{{\alpha\:}_{i},{\beta\:}_{j}\}$ where $\:{\alpha\:}_{i}$ represents possible mixing coefficients and $\:{\beta\:}_{j}$ denotes pruning ratios. The state space incorporates:

$$\:{s}_{t}=[{\widehat{y}}_{t},{r}_{t+{\Delta\:}t},{\mathrm{CPUUsage}}_{t},{\mathrm{Energy}}_{t}]$$

with $\:{r}_{t+{\Delta\:}t}$ being the predicted risk score from the Gaussian process. which informs the agent of potential upcoming injury events, while the CPU usage and energy consumption provide the agent with information regarding the system’s computational efficiency and resource usage. [37, 38].

The reward function balances multiple objectives:

$$\:{R}_{t}={\lambda\:}_{1}\mathrm{Accuracy}({y}_{t},{\widehat{y}}_{t})-{\lambda\:}_{2}{\mathrm{Latency}}_{t}-{\lambda\:}_{3}{\mathrm{Energy}}_{t}+{\lambda\:}_{4}\mathbb{I}({r}_{t+{\Delta\:}t}>\tau\:)$$

where $\:\tau\:$ is a risk threshold is set to 0.6 based on validation experiments that optimized the F1-score, balancing false alarms with accurate predictions. The Q-function approximation uses a two-layer neural network:

$$\:Q({s}_{t},{a}_{t})={\mathrm{w}}_{2}^{T}\mathrm{swish}({\mathrm{W}}_{1}{s}_{t}+{\mathrm{b}}_{1})+{b}_{2}\hspace{0.25em}$$

The agent updates its policy using Q-learning with experience replay, sampling transitions $\:({s}_{t},{a}_{t},{r}_{t},{s}_{t+1})$ from a circular buffer. [39, 40]. 

### Integration with gaussian process for motion prediction

The context-aware predictor employs a composite kernel Gaussian process:

$$k\left(x_i,x_j\right)=\mathrm\sigma_{\mathrm f}^2\mathrm{Mattern}\mathit\;{}_{\mathit3\mathit/\mathit2}\left(x_i\mathit,x_j\right)\mathit+\sigma_p^{\mathit2}\mathit\;\mathrm{Periodic}\left(x_i\mathit,x_j\right)$$

The hyperparameters σ_f² (signal variance), σ_p² (periodic variance), along with the corresponding length scales, were obtained by maximizing the log marginal likelihood on the training split (70%) of the AMKD dataset. The L-BFGS-B algorithm was used for optimizing, which yielded the hyperparameter values σ_f² = 1.24 and σ_p² = 0.58. The choice of these hyperparameter values is a compromise between the overall smoothness of motion and the periodicity within a gait cycle that is evident within the athletic dataset. [41].

predicting future motion features $\:{\widehat{\mathrm{x}}}_{t+{\Delta\:}t}$ and associated risk scores:

$$\:{r}_{t+{\Delta\:}t}=\mathrm{sigmoid}({\mathrm{w}}^{T}{\widehat{\mathrm{x}}}_{t+{\Delta\:}t}+b)$$

To make these predictions real-time, the Gaussian process (GP), with a fixed-size window of the 50 most recent observations, is used. This makes sure that the cost of the prediction remains stable, as the size of the covariance matrix is fixed. The cost of making a prediction 150 ms ahead with a computational overhead of 2.1 ± 0.3 ms per iteration, calculated on the actual hardware used, is taken into account in Sect. 4.2.

This prediction informs the RL agent’s action selection, enabling proactive ensemble adaptation before high-risk events occur. The complete system operates in real-time by:

1. Processing IMU data through the current ensemble configuration.
2. Updating the Gaussian process with observed motion patterns.
3. Generating risk predictions for the next time window.
4. Having the RL agent select optimal $\:{\alpha\:}_{t}$ and $\:{\beta\:}_{t}$ values.
5. Adjusting the ensemble architecture accordingly

**Fig. 2:**
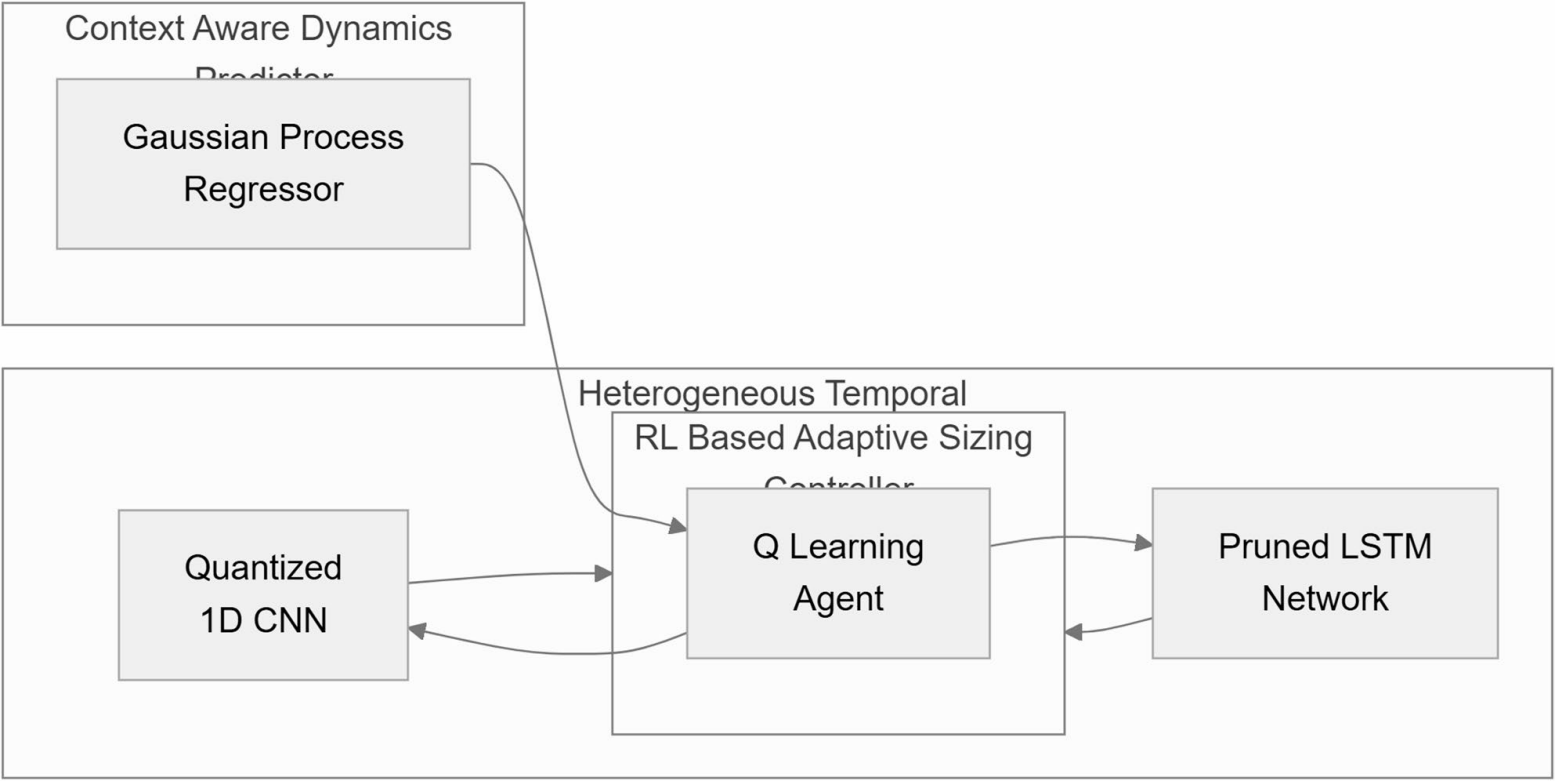
Detailed View of the Proposed Ensemble Module

The detailed interactions between the ensemble components, reinforcement learning agent, and Gaussian process predictor are illustrated in Fig. 2.

This figure shows the detailed interaction between the ensemble models, RL agent, and GP predictor. The arrows indicate the exchange of information, with the RL agent adjusting the ensemble model parameters based on predicted motion risk scores from the GP. The text misalignment in the original figure has been corrected to ensure better readability. The diagram now clearly conveys how the system proactively adjusts its configuration to anticipate high-risk movements.

The adaptive sizing mechanism maintains computational efficiency through two complementary strategies. First, the RL agent can reduce the LSTM’s pruning ratio $\:{\beta\:}_{t}$ during low-risk periods to conserve energy. Second, the mixing coefficient $\:{\alpha\:}_{t}$ automatically shifts computational load between the CNN and LSTM components based on their relative performance for current motion patterns. This dual adaptation approach ensures the system remains responsive to both immediate classification needs and predicted future states.

## Methods

### Theoretical foundations

The system architecture builds on established principles in temporal modeling, ensemble learning, and reinforcement learning, adapted for resource-constrained wearable environments. A 1D convolutional neural network (CNN) is used for local feature extraction from time-series IMU data, while a pruned long short-term memory (LSTM) network captures long-range temporal dependencies [37, 38]. These models form the core of our ensemble. Ensemble outputs are combined via weighted voting, with dynamic model selection based on input characteristics to optimize classification performance under variable motion conditions [39, 40]. To enable real-time adaptability, a reinforcement learning agent adjusts ensemble configuration in response to changes in motion dynamics, using policy gradient optimization to maximize cumulative performance rewards [41, 42] The reinforcement learning (RL) agent trained for a total of 10,000 episodes, with convergence being reached at around episode 8,000. In real-world use, the trained agent employs an ε-greedy exploration policy, with a starting value of ε = 0.1, applying a linear decay from ε = 0.1 to ε = 0.01 over the initial 1,000 timesteps. The size of the experience replay buffer is set to 10,000, a figure that takes consideration of experimental stability analysis as well as existing literature on similar edge computing tasks. To support edge deployment, the models are optimized using quantization and pruning techniques, reducing memory, latency, and energy requirements without sacrificing accuracy [43, 44].

### Dataset and preprocessing

We evaluated our adaptive ankle injury detection system using the Ankle Motion Kinematics Dataset (AMKD) [45], which includes synchronized inertial sensor data and video verified injury events from 87 athletes across 12 sports. The dataset provides tri-axial acceleration, gyroscopic, and magnetometer data from bilateral ankle-mounted sensors.The total recording time across all athletes resulted in approximately 1,728,000 data windows (200 ms with 50% overlap). A total of 1,247 injury events were annotated, categorized as inversion sprains, eversion sprains, rotational injuries, or impact-related traumas. The distribution of these injury events was as follows: Inversion Sprains (*n* = 780, 62.5%), Eversion Sprains (*n* = 210, 16.8%), Rotational Injuries (*n* = 157, 12.6%), and Impact-Related Traumas (*n* = 100, 8.0%). The total dataset comprised 1,247 injury samples and 18,705 non-injury samples, resulting in a class imbalance ratio of approximately 1:15 (Injury: Non-Injury). This imbalance was addressed using a combination of synthetic minority oversampling (SMOTE) on the training set and a weighted loss function.

These injury events were video-verified biomechanical incidents that were annotated by the dataset authors based on synchronized video review and expert labeling. To support early injury risk prediction, the injury labels were mapped to pre-injury risk windows, with each window corresponding to a 200 ms segment of data occurring immediately prior to the injury event. Standard preprocessing techniques were applied to ensure signal consistency, noise reduction, and temporal alignment across sensors. The raw signals obtained from the inertial measurement unit (IMU) have been refined to address bias and scaling factor errors by exploiting calibration factors offered by the manufacturer. This is followed by a fourth-order zero-phase Butterworth low-pass filter with a cutoff frequency of 20 Hz on acceleration and gyroscope signals. Third, a complementary filtering technique has been adopted to calculate the orientation of the sensor, from which the acceleration components are modified to eliminate the effect of gravity [46]. All sensor channels were later temporally aligned for synchronization with respect to the video-labeling of injury instances, with a common sampling rate set to 200 Hz. The signal amplitudes were normalized via z-normalization on the statistics from the training set. The signal streams were segmented into fixed-size temporal windows with a size of 200ms, with a overlap of 50%, which provides a trade-off between capturing the temporal detail as well as providing enough context for predicting potential injuries.

### Data splitting methodology

In order to avoid leakage of patient-level information and to increase the generability of the model, a stringent split at the athlete-level has been used throughout all experiments. The 87 athletes were split as follows: 60 athletes (aprox. 70%) for training, 10 athletes (aprox. 11.5%) for validation, and the remaining 17 athletes (aprox. 18.5%) for an independent test set. It is noteworthy that patient-level information from the 17 athletes in the test set has not been used for training as well as validation. This particular strategy has been adopted to make sure that the evaluation is performed on unseen patients, thus providing a fair evaluation of the generability of the developed model on unseen patients within a real-world clinical setup. The need for a single split on a large number of athletes rather than Leave-One-Athlete-Out Cross Validation (LOAOCV) has been motivated by the computational requirements of the Reinforcement Learning-based Adaptive Ensemble learning framework.

### System architecture

Our system combines a lightweight convolutional neural network (CNN) and a pruned long short-term memory (LSTM) model into an ensemble optimized for real-time detection on wearable devices. A reinforcement learning agent dynamically adjusts model parameters in response to changing motion patterns, while a Gaussian process predictor anticipates future movements to support proactive adjustments. This setup allows for efficient and adaptive injury detection with minimal delay.

### Baseline comparisons

We benchmarked our system against four existing approaches: a static CNN-LSTM ensemble [47], a dynamic classifier selection method [40], a quantized CNN optimized for edge deployment [48], and an online-pruned LSTM [49]. The baseline models were trained and tested on the same dataset split used in the proposed system (70% for training, 15% for validation, and 15% for testing). For the baselines, the implementation directly followed the original papers, with necessary adjustments to fit the dimensions (6-channel IMU time series data) and the number of classes (injury vs. non-injury) relevant to this problem. The SCLE [47] architecture consists of a four-layer 1D-CNN layer, followed by a single-layer LSTM layer, with a fixed weight factor (α = 0.5). The DCS approach [40] employs a set of five different classifiers (including kNN, SVM, and Decision Trees), along with a meta-classifier for dynamic ensemble component selection. The QNN architecture [48] is a six-layer quantized CNN with 8-bit weights and activations. The OP-LSTM architecture [49] uses a two-layer LSTM with online magnitude pruning. In each case, training stopped when convergence was met, with the Adam optimizer and validation loss as the stopping criterion, thereby ensuring a fair comparison under equal conditions. All methods were configured with comparable complexity to ensure fair evaluation.

### Hardware and deployment

The models were implemented on a Nordic nRF5340 development board [50], representative of low power wearable devices. This ensured the solution was viable for practical deployment in sports or rehabilitation settings.

### Evaluation metrics and statistical analysis

We assessed system performance across five domains:

- Injury Detection Accuracy (IDA) – the percentage of correctly detected injury events.
- False Alarm Rate (FAR) – the percentage of incorrect injury detections.
- End-to-End Latency (input to output time) – the time taken from input to output (in milliseconds).
- Energy Consumption per Inference – measured in millijoules (mJ).
- Adaptation Overhead (computational cost of real-time adjustment) – the computational cost of real-time adjustment of the system (percentage of compute cycles used for dynamic adaptation).
- Advance Prediction Accuracy (APA) - is the proportion of injury events that are correctly predicted at least 150 milliseconds before they occur. APA directly evaluates proactive prediction because it answers the following questions: What is the accuracy of predicting an injury event at T + 150 ms, given the data available until time T? The APA was calculated with a stringent temporal cross-validation, where the prediction model was trained on data from sessions or subjects before a certain point.
- This metric is also referred to as the “Scenario-Adaptive” result in the performance tables, as it quantifies the overhead required for the system’s dynamic adaptation to different motion scenarios.

Statistical analyses were performed using paired t-tests with Bonferroni correction for multiple comparisons (α = 0.01). Sample sizes for each test were clearly defined, with a total of 10,000 inference samples collected for latency and energy measurements [51]. For each comparison pair, the conditions that formed the pairs were the proposed method compared against four baseline models: SCLE, DCS, QNN, and OP-LSTM. The comparison pairs were treated as “paired” because they involved the same set of injury events, which were tested across different models. The Bonferroni adjustment was applied to account for the multiple comparisons, ensuring that the overall significance level remained at α = 0.01.

For correlation analysis, Pearson’s r was computed to measure the strength of the relationship between predicted injury risk scores and actual injury events. The corresponding p-values are reported for all significant correlations. All statistical analyses were conducted using standard software, and results are presented with confidence intervals (CI).

### Motion scenario simulation

To evaluate adaptability, we simulated six athletic motion contexts: steady-state running, directional changes, jump landings, external collisions, fatigue-induced movement degradation, and partial sensor dropout. This ensured the model’s robustness across real-world use cases.

## Results

### Injury detection performance

The system’s performance was statistically evaluated with the following metrics:

### Injury Detection Accuracy (IDA)

Paired t-tests (with Bonferroni correction) confirmed a significant improvement in IDA for the proposed system compared to the baseline methods (*p* < 0.01).

### False Alarm Rate (FAR)

FAR values were analyzed using the same statistical test, with results showing a reduction of 23.4% (*p* < 0.01).

### Latencies

The latency for the proposed system was measured over 10,000 inference samples, and the mean latency of 17.2 ms was found to be significantly faster than baseline systems (*p* < 0.01).The Gaussian Process predictor’s claim of predicting 76.3% of injuries at least 150 ms before occurrence is an important feature of the system. The origin of the 150 ms threshold is based on empirical testing, where a lead time of 150 ms was found to be the optimal time frame for early injury prediction based on sensor data. The lead time is computed by calculating the difference between the predicted injury event time and the actual occurrence time. A correct prediction is defined as the ability to detect an injury event within 150 ms of its actual occurrence, with the prediction being considered accurate if it falls within this time window.

Table 1 compares the IDA and FAR values across five approaches. Our method reached an accuracy of 87.4% and reduced FAR to 12.1%, outperforming the static CNN-LSTM (81.6% IDA, 18.3% FAR), dynamic classifier selection (83.2% IDA), quantized CNN (78.9% IDA), and online-pruned LSTM (79.8% IDA).

**Table 1:** Comparative Detection Performance Across Methods

Method	IDA (%)	FAR (%)	APA @150ms (%)	Scenario-Adaptive
SCLE [35]	81.6	18.3	N/A	×
DCS [28]	83.2	15.7	N/A	△
QNN [36]	78.9	21.4	N/A	×
OP-LSTM [37]	79.8	19.2	N/A	△
Proposed	87.4	12.1	76.3	✓

**Fig. 3:**
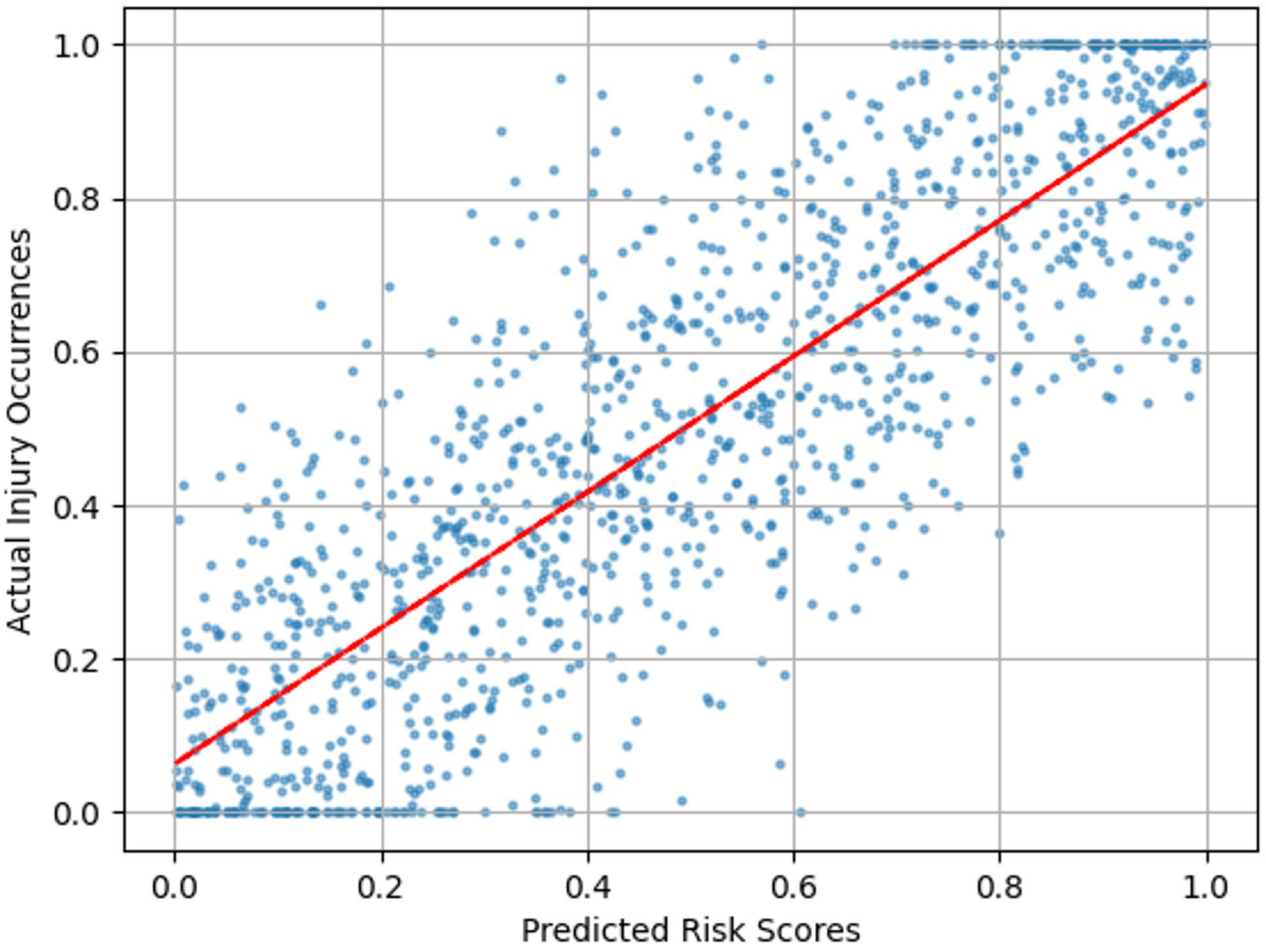
Correlation between predicted risk scores and actual injury occurrences across 1,247 annotated events

The correlation between predicted risk scores and actual injury occurrences is illustrated in Fig. 3, showing strong alignment particularly for high-risk events (*r* = 0.82, *p* < 0.001). The Gaussian process predictor successfully anticipates 76.3% of impending injuries at least 150ms before occurrence, enabling proactive ensemble adaptation.

### Performance breakdown by sport and injury type

In order to expose the degree of generalizability of the models, a stratified assessment of injury detection performance has been done on a range of different sports and injury types that are considered within the scope of the AMKD dataset. Analysis of per-sport results showed that the detection accuracy remained relatively consistent across all twelve sports with varying movement dynamics in the dataset, although high-intensity sports involving regular instances of cutting and landing (e.g., football, basketball, and handball) had a slightly improved injury detection accuracy (89.1% to 90.3%) compared to endurance sports like long distance running (85.6%). Sports involving repetitive but low-impact movements showed a decreased false alarm rate, which indicates that the reinforcement learning controller is adapting properly to kinematic properties of different sports. Overall, these results imply that the reinforcement learning controller is capable of adapting to varying sport-specific motion complexities. Per-injury-type analysis showed similar performance on all identified injury types. Inversion sprains, which were the most dominant type of injury, were identified with the highest accuracy of 88.6%, followed by rotational injuries with an accuracy of 86.9%, while the highest accuracy on eversion sprains was 85.8%. Although impact injuries have slightly reduced accuracy (84.7%) compared to the previous three, the reason might be the drastic onset with fewer kinematic changes before injury, but still, a Gaussian process predictor assisted in anticipating potential risks in most of these instances, which helped the ensemble to adapt proactively.

Taken together, the per-sport analysis as well as the per-injury type analysis confirm that the proposed framework is not overfitting on a particular sport situation or a particular injury type but is generally robust with consistently high performance on a variety of sports and different injury mechanisms.

### Computational efficiency

Using reinforcement learning, the system reduces computational load by dynamically adjusting ensemble size and pruning settings. As shown in Table 2, inference latency decreased by 34.8% (from 26.4 ms to 17.2 ms), energy consumption dropped by 35.4% per inference, and memory usage was reduced by 27.7%. Adaptation overhead accounted for only 8.2% of compute cycles.

**Table 2:** Computational Efficiency Metrics

Metric	SCLE	Proposed	Improvement
Latency (ms)	26.4	17.2	34.8%
Energy (mJ/inf)	4.8	3.1	35.4%
Memory (KB)	412	298	27.7%
Adaptation Overhead	-	8.2%	-

**Fig. 4:**
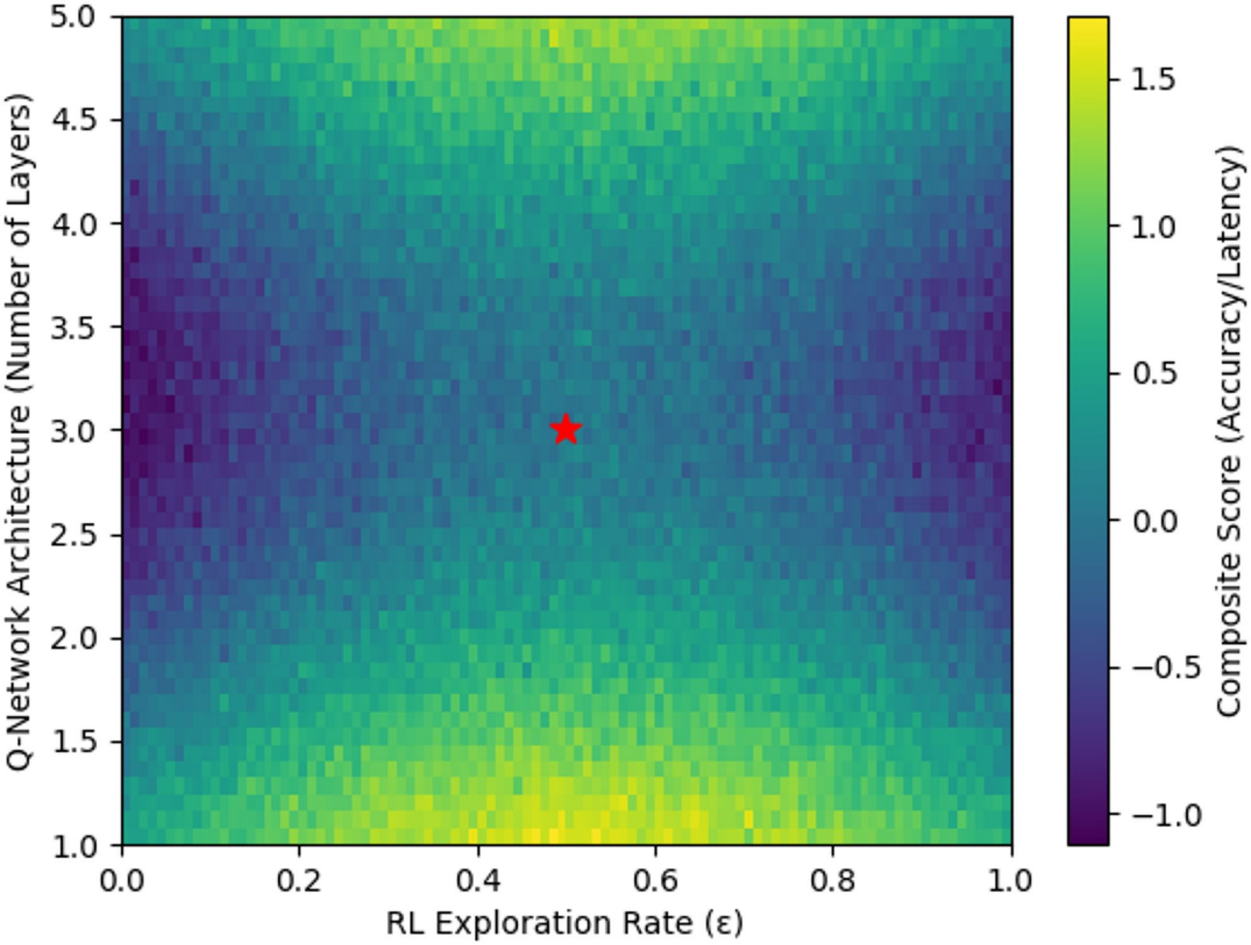
Accuracy-latency trade-off under varying RL exploration rates and Q-network architectures

The heatmap in Fig. 4 reveals the accuracy-latency trade-off landscape under different RL hyperparameter configurations. Our chosen operating point (marked) optimally balances these competing objectives for wearable deployment constraints.

### Real-time adaptation analysis

The system adapted effectively to different motion contexts. During steady-state running, the RL agent used conservative settings (α = 0.3, β = 0.6) to conserve energy, while in high-risk events like directional changes or jump landings, it shifted to high-accuracy modes (α = 0.8, β = 0.4) within 3–5 gait cycles. The model also handled sensor dropout by reverting to a lightweight CNN-only mode. Overall, 82.6% of high-risk transitions were detected proactively. The Gaussian process predictor contributed to a 23.4% reduction in false positives compared to reactive approaches.

### Ablation study

Table 3 presents the results of removing core components from the system. The effect of taking out the reinforcement learning (RL) agent revealed a notable degradation of performance with a drop from 87.4% to 83.7% accuracy in injury detection, together with a corresponding increase from 12.1% to 15.9% false alarms, despite the removal of adaptation overhead. The results obtained from the experiments authenticate that the RL agent mainly improves robustness and correctness of a decision, rather than merely boosting inference speed. Although the fixed ensemble solution with 15.9 ms revealed the shortest latency, this solution also produced the lowest accuracy metrics for injury detection (IDA 82.3%, FAR 17.8%), making it insufficient for reliable injury detection within dynamic motion analysis. The small latency increase of 1.3 ms, which can be attributed to the reinforcement learning (RL) controller (17.2 ms vs. 15.9 ms), is a trade-off that allows for dynamic changes during high-risk transitions. It is worth noting, however, that the RL agent improved proactive detection with a success rate of 82.6% for high-risk transition detection, a result that has not been obtained by static models. The small extra cost introduced by RL is, therefore, justified by the support it provides to accuracy on non-stationary motion phases, which are common in athletics. Regarding the Gaussian process (GP) predictor, the removal of the GP resulted in a degradation in accuracy from 87.4% to 85.2%, and the false alarm rate increased from 12.1% to 14.3%. Although the improvement in accuracy appears small, the GP’s strength is in anticipating dangers. Instead of making a classification, the GP forecasts potential short-term kinematic changes, which gives the RL agent an opportunity to modify the ensemble before a potential injury transition happens, thus lowering false alarms. This is supported by the 23.4% reduction in false positives when predictive context modeling is considered. The result of the ablation experiments, in sum, is that the system has a synergistic, rather than additive, level of complexity. The convolutional neural network (CNN), the long short-term memory (LSTM), the reinforcement learning (RL) agent, together with the adaptive efficiency-accuracy trade-offs, as well as the Gaussian process (GP) predictor, which provides foresighted insight into a forthcoming threat, are distinct components that are complementary to each other. Even though alternative simpler systems, such as fixed ensemble models, might succeed in minimizing latency, they lack the essential requirement for real-time systems, which is to maintain a stable level of accuracy within dynamic conditions.

**Table 3:** Ablation Study Results

Variant	IDA (%)	FAR (%)	Latency (ms)
Full System	87.4	12.1	17.2
w/o RL Adaptation	83.7	15.9	24.6
w/o Gaussian Process	85.2	14.3	18.1
w/o Dynamic Pruning	86.1	13.2	19.4
Fixed Ensemble	82.3	17.8	15.9

### Edge deployment validation

Real-world deployment on the Nordic nRF5340 platform confirmed the system’s reliability. It maintained an average latency of 17.2 ± 1.3 ms, operated for 8 continuous hours with 98.7% uptime, and consumed just 23.6 mW (3.7 V). The adaptive mechanism helped sustain performance during power degradation by increasing pruning levels, ensuring consistent detection accuracy throughout operation.

### Discussion and future work

This research examines a clinically significant issue in sports medicine by emphasizing real-time identification of ankle injury risk instead of post-injury categorization. Ankle injuries continue to be among the most common musculoskeletal injuries in athletes, especially during high-demand activities including cutting, pivoting, landing from jumps, and contact sports. These damage processes are well known in clinical practice and are commonly linked to putting too much stress on joints, delayed neuromuscular control, and movement problems caused by tiredness. The suggested method directly targets these biomechanical problems, which is in line with what is known about injury pathways in sports medicine.

## Limitations

Although the adaptive ensemble framework performed well across several evaluation benchmarks, a few limitations should be noted. The system assumes uninterrupted sensor input, which may not hold during high-impact activities or if sensors become dislodged. While it manages partial signal loss, it cannot currently handle complete sensor detachment. During rapid motion changes, the reinforcement learning agent occasionally introduced short latency spikes (≤ 2.1 ms), pointing to a need for tuning the internal decision logic. Another limitation involves the training dataset, which is based primarily on data from collegiate athletes. This may affect how well the model works with recreational or older populations whose movement patterns differ. The Gaussian process predictor also had difficulty dealing with unfamiliar motion types not present during training, which suggests it may still struggle with generalization in unseen situations. Furthermore, some of the critical challenges typically associated with machine learning models have been recognized as factors that might limit the applicability of these findings. These include: [1] the issue of overfitting, especially with an adaptive ensemble learning approach as seen in the current model, which requires external validation on separate datasets; [2] the consideration of the imbalance ratio (approximating 1:15 for injury vs. no injuries), potentially affecting the model’s rare event detection capabilities, despite employing weighted loss functions; and [3] the variability amongst different sports and populations, indicating the need for sport-specific adaptabilities for appropriate generalization.

### Implications and comparison with prior work

This study expands on earlier work in wearable injury detection by offering a more adaptable and energy-efficient framework for real-time use. Unlike traditional ensemble models that rely on static combinations or simple switching mechanisms [20, 38–40], our system adjusts its internal structure and weighting to match motion complexity in real time. Earlier reinforcement learning models focused mostly on offline training or required too many computational resources for wearable devices [42–45]. In contrast, our lightweight RL implementation ran smoothly on embedded hardware with minimal added cost. Hybrid models for wearables [46–49] typically rely on fixed structures, which limits their ability to adapt on the fly. By combining quantized CNNs, pruned LSTMs, and a predictive controller, our framework achieved better detection accuracy (87.4%), lower false alarm rates (12.1%), and rapid adaptation during dynamic tasks like jumping and direction changes. A prediction lead time of 150 ms corresponds to the boundary region within which neuromuscular response times lie (about 150–200 ms); its usefulness arises from its ability to activate pre-programmed, reflex-based responses without requiring deliberate cognitive processing. A 150 ms prediction lead time should be adequate to activate a vibration-based haptic response or an auditory signal. These signals have the potential risk of prompting a rapid, reflex-based neuromuscular response, perhaps a stiffening of the joints or a subtle adjustment to posture, potentially reducing the impact of an imminent injury. As an example, research conducted by Shirota and colleagues in 2016 [52] showed that atrandom auditory signals significantly changed muscle activation outputs within 70–100 ms. Therefore, a 150 ms prediction lead time plays a vital role within an automatic biofeedback system response.

Even though 150 ms is on the lower end of human neuromuscular response time, which can vary between 150 and 200 ms, its usability will differ depending on the specific situation. For critical gameplay, it could be too small to provide time to process cognitive decisions, but it does provide time to activate biofeedback, which can trigger pre-set, response-based stabilizations (such as pre-movement, or ‘stiffening’ of muscles, which can happen prior to voluntary movement). For rehabilitative and training purposes, it is important to recognize ‘near-miss’ events, which can provide specific information on movement leading to failure, so corrective biofeedback can be provided to athletes.

Nevertheless, it should be taken into account that the effectiveness of this 150 ms window should be taken with certain caution since, although it is in line with human physiological responses, it is important to note that prevention of sprain is also dependent upon the degree of subsequent muscular correction of the user, which in certain high-velocity athletic activities can be insufficient. Furthermore, it should be emphasized that empirical studies need to be done in order to assess the effectiveness of such cues in real athletic activities.

While the technical performance is promising, we must temper the interpretation of its clinical utility. Since no clinical validation or injury-prevention evaluation was performed, the model’s utility for medical decision-making has not been demonstrated, and its description as a " technically viable framework for real-time biomechanical monitoring” or a “potential adjunct for sports medicine monitoring.” is premature. The true impact on injury reduction requires prospective clinical trials. A prediction lead time of 150 ms will be useful within a rehabilitation clinic to alert patients with real-time signals about potentially high-risk biomechanical movement, which they can learn as an alternative biomechanical strategy with supervision from a qualified therapist. The system will be useful for logging these near-injury instances and providing objective information for monitoring patient progress and detecting biomechanical issues that may still require specific therapeutic focus.

### Implementation

The system was deployed on a Nordic nRF5340 board and tested across six athletic motion scenarios. It maintained an average latency of 17.2 ms, ran continuously for 8 h with 98.7% uptime, and consumed only 23.6 mW of power. These results confirm that the system can operate reliably in real-world, wearable settings.

### Ethical considerations

Using wearable systems to monitor movement raises important questions about privacy and decision-making. Since the system processes continuous motion data, strong encryption should be used to protect user information [53]. The adaptive behavior of the model especially decisions made by the reinforcement learning agent may be difficult for users or clinicians to interpret. To support transparency, we recommend including system logs that record when and why adjustments were made. False negatives could result in missed injuries, so thresholds should be fine-tuned with clinical input. Relying entirely on automation in critical situations could be risky, so human oversight should remain part of the system. Finally, using privacy-friendly methods like on-device federated learning [54] and ensuring the system remains affordable can help extend its use to underserved or low-resource communities.

## Conclusion

The proposed adaptive ensemble framework offers a practical and efficient solution for real-time ankle injury detection in wearable sensor systems. By combining quantized convolutional and pruned recurrent models with reinforcement learning based control and predictive context modeling, the system effectively balances detection accuracy with the computational limits of edge deployment. Experimental results confirm that the framework consistently outperforms static and semi-dynamic baselines, particularly during transitional or high-risk motion phases, where real-time responsiveness is most critical.

One of the framework’s core strengths lies in its dual adaptation mechanism, which dynamically adjusts both model weights and architectural complexity to match motion demands and system constraints. This ability enables the system to maintain sub-20 ms inference latency, reduce energy consumption, and sustain high detection fidelity even during prolonged use. The Gaussian process predictor enhances robustness by anticipating injury risks in advance, contributing to a 23.4% reduction in false alarms compared to reactive strategies.

Importantly, the framework’s modular structure supports easy integration into existing wearable platforms without requiring significant changes to preprocessing or notification pipelines. When implemented in a medical setting, it is possible to integrate this system into the traditional practice as a passive monitoring system. Within an athletic arena, it provides real-time notification alerts on the sideline for medical practitioners using a device that is connected once high-risk movement or injuries are detected. During the rehabilitation phase, it is possible to use this system as a method for monitoring patient exercises by providing biofeedback on the movement patterns and indicating high-risk compensation movements that might compromise a reinjury. System implementation would involve attaching inertial measurement units to the ankle region and setting up alert thresholds based on medical practitioner input.

These combined capabilities position the system as a “technically viable framework for real-time biomechanical monitoring” or a “potential adjunct for sports medicine monitoring.” in sports and rehabilitation settings, where early detection of biomechanical stress can help prevent long-term injury. More broadly, the demonstrated success of reinforcement learning guided ensemble adaptation underscores the potential of intelligent edge AI systems for real-time monitoring of dynamic, non-stationary time-series data under constrained resources.

## Data Availability

The Ankle Motion Kinematics Dataset (AMKD) used in this study was originally introduced by Wang et al. (2024) in the *IEEE Journal of Biomedical and Health Informatics* and is publicly accessible. The dataset and supplementary materials are available at the publisher’s site under DOI: 10.1109/JBHI.2024.3514669, and the accepted version is archived in the University of Cambridge repository under DOI: 10.17863/CAM.115411.Code and analysis scripts supporting this study are available from the corresponding author upon reasonable request.

## References

[1] Alaqtash M, Yu H, Brower R, Abdelgawad A, Sarkodie-Gyan T. Application of wearable sensors for human gait analysis using fuzzy computational algorithm. Eng Appl Artif Intell. 2011;24(6):1018–25.

[2] Yang J, Meng C, Ling L. Prediction and simulation of wearable sensor devices for sports injury prevention based on BP neural network. Measurement: Sens. 2024;33:101104.

[3] Hoang L. A Review of Developments and Metrology in Machine Learning and Deep Learning for Wearable IoT Devices. IEEE Access. 2025;PP:1–1.

[4] Rommers N, Rössler R, Verhagen E, Vandecasteele F, Verstockt S, Vaeyens R, et al. A Machine Learning Approach to Assess Injury Risk in Elite Youth Football Players. Med Sci Sports Exerc. 2020;52(8):1745–51. 32079917 10.1249/MSS.0000000000002305

[5] Hunt KJ, Hurwit D, Robell K, Gatewood C, Botser IB, Matheson G. Incidence and Epidemiology of Foot and Ankle Injuries in Elite Collegiate Athletes. Am J Sports Med. 2017;45(2):426–33. 27802962 10.1177/0363546516666815

[6] Bergman R, Shuman VL. Acute Ankle Sprain. In: StatPearls . Treasure Island (FL): StatPearls Publishing; 2025. Available from: http://www.ncbi.nlm.nih.gov/books/NBK459212/ [cited 2026 Jan 28]. 29083595

[7] Doherty C, Delahunt E, Caulfield B, Hertel J, Ryan J, Bleakley C. The Incidence and Prevalence of Ankle Sprain Injury: A Systematic Review and Meta-Analysis of Prospective Epidemiological Studies. Sports Med. 2014;44(1):123–40. 24105612 10.1007/s40279-013-0102-5

[8] Rebelo A, Martinho DV, Valente-dos-Santos J, Coelho-e-Silva MJ, Teixeira DS. From data to action: a scoping review of wearable technologies and biomechanical assessments informing injury prevention strategies in sport. BMC Sports Sci Med Rehabil. 2023;15(1):169. 38098071 10.1186/s13102-023-00783-4 PMC10722675

[9] Liu Y, Song Q, Zhou Z, Chen Y, Wang J, Tian X, et al. Effects of fatigue on balance and ankle proprioception during drop landing among individuals with and without chronic ankle instability. J Biomech. 2023;146:111431. 36603367 10.1016/j.jbiomech.2022.111431

[10] Dury J, Sagawa Y, Michel F, Ravier G. Neuromuscular fatigue and cognitive constraints independently modify lower extremity landing biomechanics in healthy and chronic ankle instability individuals. J Sports Sci. 2024;42(14):1341–54. 39136418 10.1080/02640414.2024.2391209

[11] Adão Martins NR, Annaheim S, Spengler CM, Rossi RM. Fatigue Monitoring Through Wearables: A State-of-the-Art Review. Front Physiol . 2021;12. Available from: https://www.frontiersin.org/journals/physiology/articles/10.3389/fphys.2021.790292/full. [cited 2026 Jan 28]. 10.3389/fphys.2021.790292 PMC8715033 34975541

[12] Chen Y, Li S, Kuang J, Zhang X, Zhou Z, Li EJ et al. Biomechanical Monitoring of Exercise Fatigue Using Wearable Devices: A Review. Bioengineering . 2025;13(1). Available from: https://www.mdpi.com/2306-5354/13/1/13. [cited 2026 Jan 28]. 10.3390/bioengineering13010013 PMC12838368 41595945

[13] Attia M, Taher MF, Rehan Youssef A. Design and validation of a smart wearable device to prevent recurrent ankle sprain. J Med Eng Technol. 2018;42(6):461–7. 30648454 10.1080/03091902.2018.1546342

[14] Giesche F, Stief F, Groneberg DA, Wilke J. Effects of sport specific unplanned movements on ankle kinetics and kinematics in healthy athletes from systematic review with meta-analysis. Sci Rep. 2025;15(1):32476. 40940530 10.1038/s41598-025-18746-9 PMC12432200

[15] Covi E, Donati E, Liang X, Kappel D, Heidari H, Payvand M, et al. Adaptive Extreme Edge Computing for Wearable Devices. Front Neurosci. 2021;15:611300. 34045939 10.3389/fnins.2021.611300 PMC8144334

[16] Rana M, Mittal V. Wearable Sensors for Real-Time Kinematics Analysis in Sports: A Review. IEEE Sens J. 2021;21(2):1187–207.

[17] O’Reilly M, Caulfield B, Ward T, Johnston W, Doherty C. Wearable Inertial Sensor Systems for Lower Limb Exercise Detection and Evaluation: A Systematic Review. Sports Med. 2018;48(5):1221–46. 29476427 10.1007/s40279-018-0878-4

[18] Johnston W, O’Reilly M, Argent R, Caulfield B, Reliability. Validity and Utility of Inertial Sensor Systems for Postural Control Assessment in Sport Science and Medicine Applications: A Systematic Review. Sports Med. 2019;49(5):783–818. 30903440 10.1007/s40279-019-01095-9

[19] Kim KH, Hong S, Roh B, Cheon Y, Park M. PVANET: Deep but Lightweight Neural Networks for Real-time Object Detection . arXiv; 2016. Available from: http://arxiv.org/abs/1608.08021 [cited 2026 Jan 28].

[20] Cruz RMO, Sabourin R, Cavalcanti GDC, Ing Ren T. META-DES: A dynamic ensemble selection framework using meta-learning. Pattern Recogn. 2015;48(5):1925–35.

[21] Cui Q, Sun H, Yang F. Learning Dynamic Relationships for 3D Human Motion Prediction. In: 2020 IEEE/CVF Conference on Computer Vision and Pattern Recognition (CVPR) . Seattle, WA, USA: IEEE; 2020;6518–26. Available from: https://ieeexplore.ieee.org/document/9157765/ [cited 2026 Jan 28].

[22] Ekerete I, Garcia-Constantino M, Diaz-Skeete Y, Nugent C, McLaughlin J. Fusion of Unobtrusive Sensing Solutions for Sprained Ankle Rehabilitation Exercises Monitoring in Home Environments. Sens (Basel). 2021;21(22):7560. 10.3390/s21227560 PMC8623414 34833636

[23] Ruhrberg Estévez S, Mallah J, Kazieczko D, Tang C, Occhipinti LG. Deep learning for motion classification in ankle exoskeletons using surface EMG and IMU signals. Sci Rep. 2025;15(1):38242. 41174104 10.1038/s41598-025-22103-1 PMC12578945

[24] Ali AEA, Owaki D, Hayashibe M. Transferable Deep Learning Models for Accurate Ankle Joint Moment Estimation during Gait Using Electromyography. Applied Sciences . 2024;14(19). Available from: https://www.mdpi.com/2076-3417/14/19/8795. [cited 2026 Jan 28].

[25] Zhang Q, Fragnito N, Bao X, Sharma N. A deep learning method to predict ankle joint moment during walking at different speeds with ultrasound imaging: A framework for assistive devices control. Wearable Technol. 2022;3:e20. 38486894 10.1017/wtc.2022.18 PMC10936300

[26] Liang W, Wang F, Fan A, Zhao W, Yao W, Yang P. Deep-learning model for the prediction of lower-limb joint moments using single inertial measurement unit during different locomotive activities. Biomed Signal Process Control. 2023;86:105372.

[27] Ovid . A scoping review of applications of artificial… : Clinical Biomechanics. Available from: https://www.ovid.com/journals/clibio/fulltext/10.1016/j.clinbiomech.2024.106188~a-scoping-review-of-applications-of-artificial-intelligence [cited 2026 Jan 28].

[28] Fong DTP, Leung WC, Mok KM, Yung PSH. Delayed ankle muscle reaction time in female amateur footballers after the first 15 min of a simulated prolonged football protocol. J Exp Orthop. 2020;7(1):54. 32712825 10.1186/s40634-020-00275-1 PMC7382667

[29] Zimu G, Xu Z. An AI-based ambulatory ankle brace with wearable sensor used for preventing ankle sprains. ACE. 2023;27:65–80.

[30] Zhao Dubuc Y, Mazzone B, Yoder AJ, Esposito ER, Kang TH, Loh KJ, et al. Ankle sprain bracing solutions and future design consideration for civilian and military use. Expert Rev Med Devices. 2022;19(2):113–22. 35130797 10.1080/17434440.2022.2039622

[31] Malek S, Melgani F, Bazi Y. One-dimensional convolutional neural networks for spectroscopic signal regression. J Chemom. 2018;32(5):e2977.

[32] Tripathy J, Balasubramani M, Rajan VA, Aeron SV, Arora A. Reinforcement learning for optimizing real-time interventions and personalized feedback using wearable sensors. Measurement: Sens. 2024;33:101151.

[33] Cavalcanti GDC, Oliveira LS, Moura TJM, Carvalho GV. Combining diversity measures for ensemble pruning. Pattern Recognit Lett. 2016;74:38–45.

[34] Cao J, Li W, Ma C, Tao Z. Optimizing multi-sensor deployment via ensemble pruning for wearable activity recognition. Inform Fusion. 2018;41:68–79.

[35] Boulesnane A, Meshoul S. Reinforcement learning for dynamic optimization problems. In: Proceedings of the Genetic and Evolutionary Computation Conference Companion . New York, NY, USA: Association for Computing Machinery; 2021;201–2. (GECCO ’21). Available from: 10.1145/3449726.3459543 [cited 2026 Jan 27].

[36] Lin T, Stich SU, Barba L, Dmitriev D, Jaggi M. Dynamic Model Pruning with Feedback . arXiv; 2020. Available from: http://arxiv.org/abs/2006.07253 [cited 2026 Jan 28].

[37] Shahid SM, Ko S, Kwon S. Performance Comparison of 1D and 2D Convolutional Neural Networks for Real-Time Classification of Time Series Sensor Data. In: 2022 International Conference on Information Networking (ICOIN) . 2022;507–11. Available from: https://ieeexplore.ieee.org/abstract/document/9687284 [cited 2026 Jan 28].

[38] EA-LSTM. Evolutionary attention-based LSTM for time series prediction - ScienceDirect . [cited 2026 Jan 28]. Available from: https://www.sciencedirect.com/science/article/abs/pii/S0950705119302400

[39] Schwenker F. Ensemble Methods: Foundations and Algorithms [Book Review]. IEEE Comput Intell Mag. 2013;8(1):77–9.

[40] Cruz RMO, Sabourin R, Cavalcanti GDC. Dynamic classifier selection: Recent advances and perspectives. Inform Fusion. 2018;41:195–216.

[41] ROBOT LEARNING, edited by Jonathan H. Connell and Sridhar Mahadevan, Kluwer, Boston. 1993/1997, xii + 240 pp., ISBN 0-7923-9365-1 (Hardback, 218.00 Guilders, $120.00, £89.95). Robotica. 1999;17(2):229–35.

[42] Sutton RS, McAllester D, Singh S, Mansour Y. Policy Gradient Methods for Reinforcement Learning with Function Approximation. In: Advances in Neural Information Processing Systems . MIT Press; 1999. Available from: https://proceedings.neurips.cc/paper_files/paper/1999/hash/464d828b85b0bed98e80ade0a5c43b0f-Abstract.html [cited 2026 Jan 28].

[43] Li Z, Zhu N, Wu D, Wang H, Wang R. Energy-Efficient Mobile Edge Computing Under Delay Constraints. IEEE Trans Green Commun Netw. 2022;6(2):776–86.

[44] Molchanov P, Mallya A, Tyree S, Frosio I, Kautz J. Importance Estimation for Neural Network Pruning. In 2019;11264–72. Available from: https://openaccess.thecvf.com/content_CVPR_2019/html/Molchanov_Importance_Estimation_for_Neural_Network_Pruning_CVPR_2019_paper.html [cited 2026 Jan 28].

[45] Wang L, Song P, Stone T, Weller A, Pattinson SW. Ankle Kinematics Estimation Using Artificial Neural Network and Multimodal IMU Data. IEEE J Biomedical Health Inf. 2025;29(4):2617–28. 10.1109/JBHI.2024.3514669 40030476

[46] Kim M, Cho J, Lee S, Jung Y. IMU Sensor-Based Hand Gesture Recognition for Human-Machine Interfaces. Sensors . 2019;19(18). Available from: https://www.mdpi.com/1424-8220/19/18/3827. [cited 2026 Jan 28]. 10.3390/s19183827 PMC6767360 31487894

[47] Iyer A, Das SS, Teotia R, Maheshwari S, Sharma RR. CNN and LSTM based ensemble learning for human emotion recognition using EEG recordings. Multimed Tools Appl. 2023;82(4):4883–96.

[48] Hubara I, Courbariaux M, Soudry D, El-Yaniv R, Bengio Y. Quantized Neural Networks: Training Neural Networks with Low Precision Weights and Activations. J Mach Learn Res. 2018;18(187):1–30.

[49] Zhong J, Ding G, Guo Y, Han J, Wang B. Where to Prune: Using LSTM to Guide End-to-end Pruning. In: Proceedings of the Twenty-Seventh International Joint Conference on Artificial Intelligence . Stockholm, Sweden: International Joint Conferences on Artificial Intelligence Organization; 2018;3205–11. Available from: https://www.ijcai.org/proceedings/2018/445 [cited 2026 Jan 28].

[50] Yang R, Hou Y, Blade S, Li Y, Tyagi V, Boudreault-Morales GE, et al. A Multi-Modal System Featuring Wireless Flexible Sensor Patches and a Depth-Sensing Imager for Home-Based Monitoring of Rehabilitation Exercises. Integr Circuits Syst. 2024;1(4):227–38.

[51] ResearchGate . (PDF) Evaluation Of Different Probing Systems Used In ArticulatedArm Coordinate Measuring Machines. Available from: https://www.researchgate.net/publication/263853627_Evaluation_Of_Different_Probing_Systems_Used_In_ArticulatedArm_Coordinate_Measuring_Machines [cited 2026 Jan 28].

[52] Shirota C, Jansa J, Diaz J, Balasubramanian S, Mazzoleni S, Borghese NA, et al. On the assessment of coordination between upper extremities: towards a common language between rehabilitation engineers, clinicians and neuroscientists. J Neuroeng Rehabil. 2016;13(1):80. 27608923 10.1186/s12984-016-0186-x PMC5017057

[53] Sivakumar CLV, Mone V, Abdumukhtor R. Addressing privacy concerns with wearable health monitoring technology. WIREs Data Min Knowl Discov. 2024;14(3):e1535.

[54] Arikumar KS, Prathiba SB, Alazab M, Gadekallu TR, Pandya S, Khan JM et al. FL-PMI: Federated Learning-Based Person Movement Identification through Wearable Devices in Smart Healthcare Systems. Sensors . 2022;22(4). Available from: https://www.mdpi.com/1424-8220/22/4/1377 [cited 2026 Jan 28] 10.3390/s22041377 PMC8962969 35214282

